# Real-world experience of the clinical effectiveness of omalizumab use in Qatar – a study protocol

**DOI:** 10.5339/qmj.2022.fqac.3

**Published:** 2022-03-28

**Authors:** Saleema Purayil, Hassan Mobayed, Maryam Al-Nesf

**Affiliations:** ^1^Allergy & Immunology Division, Department of Medicine, Hamad Medical Corporation, Qatar E-mail: SPurayil3@hamad.qa

**Keywords:** clinical practice, omalizumab, real-life effectiveness

## Abstract

Omalizumab (XOLAIR®) is a recombinant DNA-derived humanized IgG1κ monoclonal antibody that binds to IgE and was first introduced in Qatar in 2009. Omalizumab is used to treat moderate-to-severe allergic asthma (SAA), chronic idiopathic urticaria (CIU), and chronic rhinosinusitis with nasal polyposis (CRSwNP). In this study, we have described a proposal to investigate the outcomes and impact of the clinical use of omalizumab for the labeled indications (SAA, CIU, and CRSwNP) and other off-label indications (food allergy, dermatitis, and others) in clinical practice in Qatar. This is a mixed-design study in which the first stage included a chart review of all the patients from the Allergy and Immunology Division registry who received omalizumab since May 2009. The second stage was a cross-sectional questionnaire to review all (previous and current) patients and identify their current health status and treatment outcomes. The third stage was a proof of concept and consisted of selecting a cohort to investigate the omalizumab mechanism for patients before and after administration. Patients with a physician diagnosis of asthma and/or urticaria fulfilling the diagnostic criteria for omalizumab administration and patients with off-label indications were recruited. Expected outcomes included identifying the real-life effectiveness and the experience of using omalizumab in Qatar and reporting all potential adverse effects that might have been missed during early trials or other published studies. This study was essential to observe Qatar’s local clinical practice regarding the use of omalizumab; and in addition, we could gather data about the risk factors contributing to disease recovery or progression and those resulting in favorable or unfavorable outcomes. We expect the results to augment the current knowledge about understanding diseases and the global experience on the use of omalizumab.
